# Developing Inclusive Community Platforms: A Catalyst for DEI Community

**DOI:** 10.12688/openreseurope.17653.1

**Published:** 2024-06-05

**Authors:** Hiroko Costantini, Misato Nihei, Takazumi Ono, Kai Tanabe, Yoichi Motomura

**Affiliations:** 1 Oxford Institute of Population Ageing, University of Oxford, 66 Banbury Road, Oxford, England, OX2 6PR, UK; 2 Institute for Future Initiatives, The University of Tokyo, Tokyo, 7-3-1 Hongo Bunkyo-ku, 113-0033, Japan; 3 Institue of Gerontology, The University of Tokyo, 7-3-1 Hongo, Tokyo, 113-8656, Japan; 4 Graduate School of Information Science and Technology, The University of Tokyo, 7-3-1 Hongo, Tokyo, 113-8656, Japan; 5 Institute of Health and Sport Sciences, University of Tsukuba, 3 Amakubo, Tsukuba City, 305 0005, Japan; 6 Artificial Intelligence Research Center, National Institute of Advanced Industrial Science and Technology, 2-3-2 Aome, Tokyo, 135 0064, Japan

**Keywords:** DEI; Population Approach; Inclusive community; Inclusivity Indicator; Digital Twins; Well-being; Narratives; Service design

## Abstract

Developing inclusive communities is important to enhance individuals’ well-being yet this brings the challenge of actively engaging and leveraging the diversity of residents in communities. Such significant social challenges are prominent in Japan, a focus of this article, as the most advanced aging society in the world and thus relevant to European and other countries. This paper explains a major government initiative that takes an innovative approach through leveraging a social technology, a Digital Twin of a community, to understand and address inclusiveness of a community leveraging population approach. The purpose of this letter is to provide researchers and policy makers insights into the approach taken to stimulate reflection on the potential for adaptation and replication.

## Introduction

Developing inclusive communities is important to enhance individuals’ well-being yet this brings the challenge of actively engaging and leveraging the diversity of residents in communities. Issues related to social isolation and other distortions in interpersonal connections have, however, been exacerbated by societal transformations, including: weaker ties with family and neighbours, urbanization, rise of digital communication technologies, and demographic ageing. Such significant social challenges are prominent in Japan, which this article focuses on. Japan is the most advanced aging society in the world due to the increased life expectancy and declining birth rates. Therefore, its experiences and societal challenges provide relevant examples for Europe and other countries worldwide, as they may face similar issues and situations in the near future. Hence the purpose of this letter is to introduce a new research initiative that is developing a novel approach, in order to make the research project’s intent and key elements visible and thus a showcase for researchers, policymakers, and other stakeholders with a view to possible adaptation and replication.

**Figure 1.**
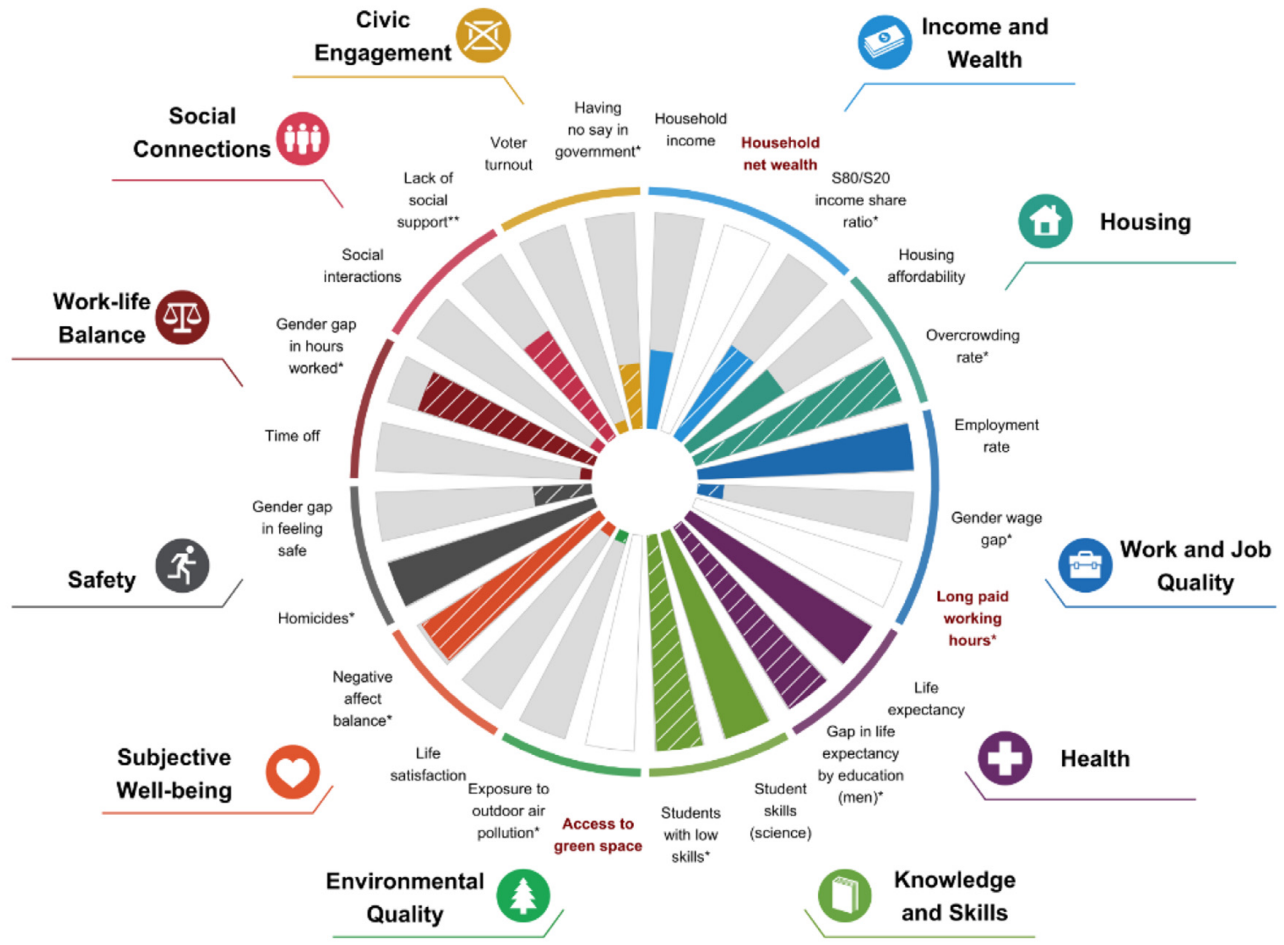
Japan's current well-being, 2022 or latest available year. Source: Helliwell, J. F., Layard, R., Sachs, J. D., Aknin, L. B., De Neve, J.-E., & Wang, S. (Eds.). (2023). How’s Life in JAPAN? World Happiness Report 2023 (11th ed.). Sustainable Development Solutions Network.

While Japan as a society has traditionally placed a high value on human relationships ( Davies & Ikeno, 2002; Doi, 1973; Hendry, 2003), Japan has the highest proportion amongst 20 OECD member countries of individuals reporting little to no interaction with people outside of their families, at 15.3% ( OECD, 2005). Correspondingly, in The World Happiness Report 2019, Japan ranked the lowest among G7 countries in terms of happiness levels, and 32nd out of 36 OECD member countries. While objective indicators in the survey such as GDP and average healthy life expectancy rank relatively high, subjective measures such as “freedom to make life choices” ranked 65th and “generosity” ranked 144th. Furthermore, the level of happiness among the Japanese people has declined over the past decade ( Helliwell *et al.*, 2023; Figure 1). To contribute to addressing these issues, in 2023 the Japanese government funded a five-year major Strategic Innovation Programme on “Developing Inclusive Community Platforms”. One of the four sub-themes ^i^ of the programme, the project this article focuses on, taking an innovative approach through leveraging a social technology, a Digital Twin of a community, to understand and address inclusiveness of a community.

In order to enhance the well-being of all community members, the project adopts a population approach addressing the community as a whole. This is in contrast to a high-risk approach that aim to reliably identify and protect individuals at high risk, as this is viewed as being effective for tax-funded policies ( Farrington, 1998; Moffitt, 1993). The high-risk approach has a significant challenge, however, in limiting engagement with non-high risk residents, which comprise the vast majority of the local population. This project, therefore, places a significant emphasis on reaching out to unengaged and indifferent groups in regard to the social issues explained above. These groups are numerous yet not fully included in the community. Therefore, the project prioritizes their voices, stories, and perspectives through *narrative*-based population approach ( Cabinet Japan, 2023).

Furthermore, narrative interviews complemented by insights derived from a systematic review of 1,649 academic papers revealed the necessity to clarify what is central to achieving inclusivity in society today. For instance, in the case of Japan, a major group to engage are older adults, particularly those who are socially isolated such as due to more limited physical and cognitive capabilities, whereas another smaller group who often feel isolated are parents of young children ( Cabinet Japan, 2023). In terms of the notion of inclusion, two key aspects that emerged were centred on tolerance and autonomy ( Galston, 2005; Raz & Mendus, 1988).

The social impact this project aims for is to maximize the diverse well-being of each individual and realize inclusive communities. To enhance the inclusiveness of communities, key to the approach taken is the development and implementation of a platform that utilizes “social technologies” grounded in social science and digital technologies. The envisioned inclusive community platform here refers to “a sustainable package of services and businesses designed to support the development of an inclusive community. These services are made accessible for selection and use (through adoption or purchase) by residents – the primary agents of the community – as well as by local governments, companies and NGOs engaged in community development. This is based on each individual’s circumstances, the happiness they aim for, and the unique conditions of their communities” ( NIBIOHN, 2023). Yet, the social technologies that would enable a transformation in values and behaviours in a community to enhance DEI have not yet been established. Nor are the human resources pivotal to active engagement within a community sufficiently fostered. Additionally, there is a lack of technical infrastructure for utilizing data that could contribute to preventing social isolation and maximizing well-being, making it difficult to assess, systematize knowledge, and replicate successful models. Furthermore, people’s highly specialized needs increase the challenge to achieving viable and sustainable initiatives (c.f., Cabinet Japan, 2023).

## Approach

The project is pursued through an interdisciplinary-based Action Research Method. To enhance the inclusiveness of communities and achievement of equity for their diverse members, a fundamental shift in people’s attitude and behaviours is crucial, such as in relation to tolerance and autonomy. Achieving such transformation requires leveraging a broad spectrum of “comprehensive knowledge” - not only to rely on scientific and technological knowledge but also encompassing humanities and social sciences. Such “comprehensive knowledge” facilitates the development of social technologies capable of influencing people’s attitudes and behaviours ( Cabinet Japan, 2023, p.8). Therefore, this project adopts an action research method, not limited to academic contributions but rather aiming for a profound societal impact. Multiple cycles of development, validation, and improvement across various communities and municipalities aim to establish social technologies that enhance inclusion for all generations ( c.f., Akiyama, 2015).

**Figure 2.**
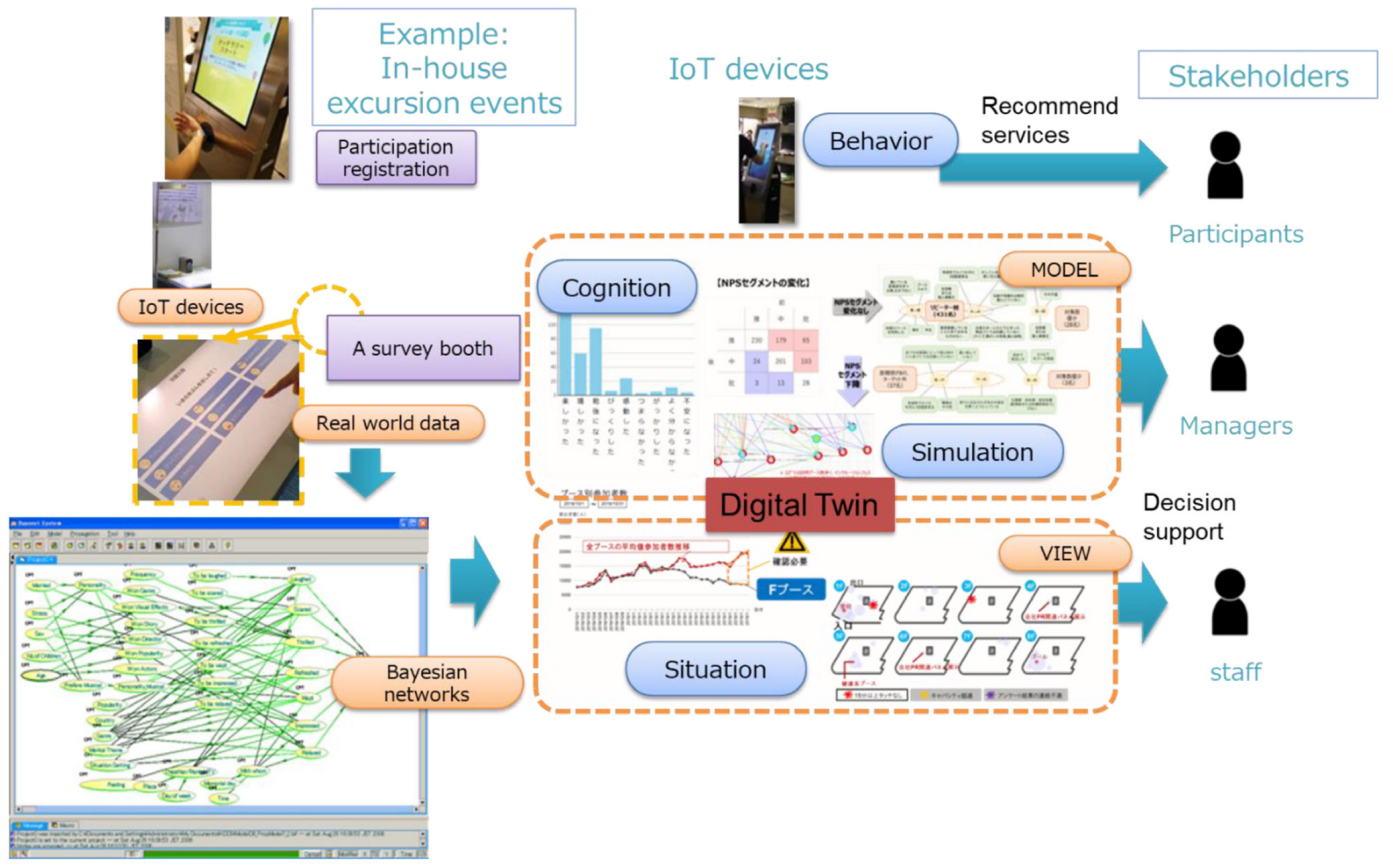
Life User Digital Twin.

Specifically, the following three main research actions are being pursued:

1.       Life User Digital Twin:

This project envisions a Life User Digital Twin ( Figure 2) as a means to externalize and integrate individual values and preferences, making them interpretable and analysable at the community level. The integration of personal data into the Digital Twin needs to achieve high privacy protection. The Digital Twin is constructed by partial aggregation (micro-aggregation) across similar people: this allows the inputs collected at individual level, such as from surveys, to be represented as probabilistic relations to outcome variables of interest. The resulting Bayesian network graph model ( Pearl, 1998) enables estimation of the probability of behaviour change in response to an intervention under certain conditions. The development of such a Digital Twin reflects learnings from past initiatives aimed at improving inclusivity. Initiatives need to respect the roles, situations, values, and intrinsic motivations of both community service users and providers, while also enabling reflection and reframing of values within collaborative projects that lead to behavioural changes. Additionally, platforms that mediate interactions between users and providers need to foster participation and facilitate mutual engagement, with support tools to use the platform designed for wide access. Such platforms have the potential to overcome typical inhibiting factors to use of appropriate services. This would include providing timely recommendations and information on appropriate services for an individual. Also, the platform would support matching of participants to appropriate activities, such as an alignment between participant’s literacy level and the difficulty of the activity, which reduces participant anxiety or boredom. Through such mechanisms the Digital Twin would support overcoming barriers to participation, which has also been identified as a common success factor.

2.       Inclusivity Indicators and Evaluation System:

A key challenge is to quantify and visualize people’s social isolation and individual well-being in daily life, which hinders analysis of the current situation and limits development of policies based on data. The project considers both academic insights and empirical evidence from field sites to conceptualize indicators to assess inclusivity. This entails formulating hypotheses for intermediate indicators (Key Performance Indicators, KPIs) and success factors (Key Factors for Success, KFSs) capable of capturing the transformation of inclusivity during interventions. The indicators will be validated through statistical analysis of ongoing evaluations and verifications in multiple areas within a research field, and comparative assessments across multiple regions, as well as consulting experts.
Correspondingly, the project will create an evaluation system to assess inclusivity in each community using statistical data (quantitative surveys), narrative elements (qualitative data such as dialogue, storytelling, and episode-based approaches) as well as data from use of the Digital Twin: this variety of data will be assessed through a relevant set of indicators from the KFS-KPI library. Furthermore, the system incorporates a “logic model”. This model establishes a causal link between KFS, KPI, and inclusivity. It is based on academic research and empirical knowledge from field practitioners. The logic model, pooled and updated by both researchers and practitioners, is integrated into the system. It aims to guide users, based on the evaluation results, towards effective practices, such as policy implementation and social actions, to promote inclusivity. While there are indicators commonly used internationally, the questions underlying those indicators remain fixed regardless of societal changes or location (e.g., WHO-5 Well-being Index, Psychological Wellbeing Scale, The Warwick-Edinburgh Mental Wellbeing Scales). This, however, is less suitable for an action-research approach that across diverse communities aims for impact through different sets of initiatives. Therefore, to enhance the versatility and applicability of the indicators, this project will also develop a tool that automatically generates appropriate indicators for each use case and thus enables calculation of the inclusivity of the community. Thus, this process will enable assessment of inclusivity over time and space: community-level indicators may be aggregated, say to regional level, and compared across communities and over time. The aim is to leverage the latest technologies such as AI to visualize “inclusivity”, so as to support the devising and evaluation of policies.

3.       Guideline development through human resource development and service demonstration:

The project aims to serve as the core for transforming people’s values and behaviours. A focus will be to develop and implement methodologies and supporting tools for finding and fostering individuals who play a core role in communities to enhance transformation of values and societal tolerance. These tools include evaluation of intervention outcomes, factor analysis, and assistance in developing improvement plans based on real world data collected locally. Also, a priority will be to promote the development of human resources amongst local government employees, local professionals, and corporate staff with the capability to effectively utilize data and the tools.
The social implementation of the Digital Twin, inclusivity assessment indicators and evaluation systems, and AI-enabled visualisation and analysis will be across diverse communities. Thus, town planning guidelines will be developed for diverse regions nationwide, encompassing human resources development methods and operational strategies such as for use of community spaces or under-utilised land or facilities.

## Discussion

The specific challenges faced by communities vary depending on their characteristics, from urban areas to depopulated areas. For instance, in Japan a growing number of communities face declining populations of both labour force and residents, though other communities face other challenges. Overcoming this fragmentation of needs is important to enable providers of services, public and private to operate effectively and sustainably. Through collaboration between industry, government, and academia, the aim is to develop an AI-based Digital Twin technology platform that in each community/region enables residents, local governments, and community-oriented businesses to select and utilize the necessary services. Furthermore, the common platform design enables recommendation of exemplary initiatives and services based on similarity across communities assessed through the rich data integrated into the Digital Twins. That is, the platform will develop and standardize methods for community planning and fostering public-private partnership businesses. This enables the optimization of initiatives and budget allocation, as well as estimation of the potential value and cost-effectiveness of initiatives and business ventures across communities.

Moreover, many collaborative projects involving industry, government, and academia tend to see initiatives and activities in the region gradually wind down once the project concludes, even if they employ action research methodologies. In this project, however, to implement the aforementioned results into society, we are designing an intermediary organization, acting as a liaison between residents, communities, businesses and local governments. Furthermore, these intermediary organizations, acting as platforms in various regions, will support local communities and engage in data-driven businesses utilizing the Digital Twin technology constructed from local data. Beyond the conclusion of the project, the aim is for core companies providing community services to continue to roll out services nationwide through the inclusive community platform. In turn, this should support inclusive communities with high sustainability to autonomously sustain themselves going forward. This approach fosters societal impact through sustainable transformation by enhancing the efficiency of community services, enriching well-being, and nurturing lasting inclusivity for the future generations.

## Ethics and consent

Ethical approval and consent were not required.

## Disclaimer

The views expressed in this article are those of the authors. Publication in Open Research Europe does not imply endorsement of the European Commission.

## Data Availability

No data are associated with this article.
