# Supplementary material for: Developing Inclusive Community Platforms: A Catalyst for DEI Community
Source: Open Res Eur. 2024 Jun 5;4:111. [Version 1] doi: 10.12688/openreseurope.17653.1 (PMC11415760; doi:10.12688/openreseurope.17653.1)
Supplement: Peer review history

# Reviewer report: Reviewer response for version 1

**DOI:** 10.21956/openreseurope.19075.r41724

**Published:** 2024-09-20

**Authors:** Wolfram Manzenreiter

**Affiliations:** 1 University of Vienna, Vienna, Austria

**Who is doing what and how when modeling DEI digital twins in Japan?**

 The Open Letter showcases the ambitions and goals of a novel AI-based approach to invigorate community functions and preserve residents’ well-being in Japan. As sub-project of a larger five-year cross-ministerial strategic innovation programme (CAO 2024a https://www8.cao.go.jp/cstp/gaiyo/sip/sipgaiyou.pdf), the ‘interdisciplinary action research’ project sets out to develop a ‘Community Digital Twin’ as technological intermediate for next-generation city planning and index- building to evaluate inclusiveness at the local level. The implementation of a Digital Twin to manage inclusion at the local level of a given community reflects the techno-optimistic vision of a ‘super-smart society’ in which social issues as well as economic growth are efficiently handled by ‘leveraging ICT to its fullest’ in merging cyberspace and physical space. The systematic integration of digital and real life worlds, initially promoted by the Japanese government’s Fifth Science, Technology and Innovation Basic Plan (2016-2020) and further adapted in its sixth instalment, has been popularized by the catchy phrase of ‘Society 5.0’ (CSTI 2015 www8.cao.go.jp/cstp/kihonkeikaku/5basicplan_en.pdf).

 As the authors point out, social isolation has become a pressing issue all over Japan. Population shifts into metropolitan conglomerates, paired with low fertility and demographic aging, and the amenities of a well-organized consumer society have propelled the erosion of family and community connections, the two main pillars of social belonging in any society, and arguably more so in Japan (Ronald and Alexy 2011)[ref 6]. Municipalities throughout the country nowadays are faced with the challenges of maintaining community coherence and functionality, despite the rapid decline in population size, tax income, and infrastructure investments (Lützeler 2018)[ref 5].

 The Open Letter singles out senior citizens and single parents as particularly susceptible to social isolation. Further high-risk populations would have to include disabled people, sexual minorities, and foreign migrants, sharing vulnerability caused by lack of acceptance, tolerance, and integration. However, survey data point out that social disconnect has arrived in the mid of society: Only 17.9% of a nation-wide sample reported in 2023 that they did not feel lonely, while more than 40% had at least occasionally or even frequently experienced feelings of loneliness. The prolonged feeling of isolation was particularly pronounced among younger age cohorts (CAO 2023 https://www.cao.go.jp/kodoku_koritsu/torikumi/zenkokuchousa/r5/pdf/tyosakekka_point.pdf.). Other distressing indicators include suicide mortality, which has been on the rise again, particularly during the years of the pandemic, and the increase in lonely death incidences. For 2023 statistics reveal that in 8,695 cases, the deceased remained unnoticed for a substantial number of days or even weeks (average was 18 days; SSI 2024 www.shougakutanki.jp/general/info/kodokushi/news/kodokusiReport_8th.pdf).

 Developing new policies to combat such grievances are without doubt highly needed, given that for a long time and until very recently both public and private sector agents have been reluctant in embracing diversity and treating minorities fairly without insisting on adaptation to mainstream society as a prerequisite for social inclusion. As late as in April 2024 a law went into effect aiming to advance measures to address loneliness and isolation. While Japan’s cabinet is in charge of defining a priority plan and coordinating policies at the national level, local governments are held responsible for establishing and implementing policies of prevention and countermeasures (CAO 2024b https://www.cao.go.jp/kodoku_koritsu/torikumi/jutenkeikaku/pdf/jutenkeikaku_honbun.pdf) in line with the fundamental principles of a society in which ‘not a single person suffering from loneliness and isolation is left behind; and individuals support and connect with each other’ (CAO 2024c www.cao.go.jp/kodoku_koritsu/torikumi/pdf/outline.pdf). The question is if and how these cities, towns and villages are eventually going to benefit from their digital twin, as the programme introduced in the Open Letter suggests. The answer remains to be seen, not least because of the many unknowns behind the methodological foundations of an approach that attempts to model a community twin on the basis of ‘comprehensive knowledge’ generated from residents’ narrative accounts, statistical survey data, a meta-analysis of a large body of academic papers, and insights from the use of the digital twin across multiple cycles of computation and validation.

 Ultimately the project assumes that social technologies, informing and improving the intermediary digital twin in response to real world data, will be capable of identifying key performance indicators and benchmarking key factors for success, and that the case-specific policy interventions will transform people’s values and thus their behaviour to enhance inclusivity. To put all this into practice, fostering awareness, knowhow and commitment among community leaders and local government employees is crucial. The acknowledgement of diversity among regions and communities, requiring case-sensitive solutions for different communities, the use of qualitative research methods to build on local stakeholders’ knowledge are all to be applauded but exactly how this is going to be put into practice remains an open question. I think it is unfortunate that the Open Letter is not outspoken on the relaization of the innovative research strategy (e.g. choice of research site/s and the theoretical assumption of how social capital, clinical data and platform utilization are supposed to increase inclusion), and I think it is unnecessary because the authors are not merely commenting on a political initiative they happened to be intrigued by but introducing the project they apparently are commissioned to steer.

  For a critical review, readers would need to know far more about the operationalisation, on the one hand, and the foundations on the other. The general lack of critical reflection and transparency is letting readers wonder about the theoretical assumptions of individual well-being and community inclusion, the tricky art of quantifying qualitative information from narrative interviews for the digital model, the level of complexity the project aspires to model, and how the digital twin is expected to deal with the intrinsic unpredictability of social systems where human behaviour is critical to their structure and dynamics (Batty 2024)[ref 1].

 Some people might think communities, social interaction and subjective well-being are easy to explain while in fact none of them are. My research group can attest to the methodological challenges in assessing well-being (Holthus and Manzenreiter 2020)[ref 4], the substantial weight of personality factors in experiencing subjective well-being, which even the most detailed clinical patient database fails to report and thus hardly ever surfaces in statistical analysis (Askitis 2023 ubdata.univie.ac.at/AC17194063), whereas social capital did not prove to be a reliable predictor for community affairs and well-being (Ueno, Ōsuga and Manzenreiter 2020[ref 7]; Askitis and Hundsdorfer forthcoming https://japanologie.univie.ac.at/en/socialcapital/). We may assume that in the case of this project communities largely conflate with municipalities, its sub-districts or even neighbourhoods. But the administrative definitions are often at odds with the lived reality of people on the ground, particularly after the Heisei merger wave from twenty years ago (Ganseforth and Jentzsch 2021)[ref 2]. So who (and when) decides about borders and boundaries when it comes to measure inclusion, and who or what defines the parameters to quantify inclusion? As inclusion here is a policy problem definition, the digital model designed to address it is very much contingent on political ideals or ideology and the expertise political elites are putting their trust in.

 Populations and their activities are shaped by a myriad of processes, and any attempt to remodel them is causing sheer unsurmountable problems for data collection and interpretation, computation time and algorithmic accuracy for taking third- or more-order interaction factors into account. Without a thorough theory, that is a solid understanding of causality which factors in the convoluted relationships among a seemingly endless number of aspects and agents within a social system, its sub-systems and the larger ecology of similar type of systems, Data analysis can sometimes be less reliable than expected when it comes to predict the effects of political intervention (Wan et al. 2024)[ref 3].

 Nothing of this is discussed or even mentioned in the Open Letter, which is not just due to space limitations, the special format of this communication or the distance between researchers intrigued by an innovative government-sponsored initiative and the project itself. A bit of snowballing investigation following the money from the hints offered in the footnotes revealed far more about the relationships between the commissioning agency, executing research institutions, their contracted partners (including one of Japan’s leading commercial urban and residential area developer specialised in building, contracting and managing real estate property) and the Open Letter authors (CAO 2024d www8.cao.go.jp/cstp/gaiyo/sip/sip_3/keikaku/03_community.pdf, AIST 2024( housetsu-community-a1.jp); TWR 2024 www.twr.jp/). One of the lead authors from the Artificial Research Center at the National Institute of Advanced Industrial Science and Technology serves as project director, and all others are at least affiliated with research institutions that are contributing to the project. If not for ethical reasons, then for academic clarity I would welcome to see the authors utilize the iterant potential of this publication format to reflect or comment on the blanks and open questions addressed in this review.

 Finally, a digital twin is only as good as the data it is drawing on. Given the long history of applied urban modelling and the way it failed so far to solve some of the most pressing problems of community life. such as ecologically efficient land use, affordable housing, and social disparities, it would be wise to see the value of the digital twin not so much in problem-solving but on focussing the awareness of researchers and politicians to ask the right kind of questions about how to address a political problem. Big data, augmented by real-time observations, can generate insights into patterns or trend developments, which is not bad at all. Yet without a solid theoretical foundation, can a modelling approach relying solely on post-hoc data explanation be good enough for sound policy recommendation, given the fast changes and ever-increasing complexity in our social life-worlds and the hard-to-predict impact of new technologies to come?

Where applicable, are recommendations and next steps explained clearly for others to follow? (Please consider whether others in the research community would be able to implement guidelines or recommendations and/or constructively engage in the debate)

Not applicable

Does the article adequately reference differing views and opinions?

No

Are all factual statements correct, and are statements and arguments made adequately supported by citations?

Partly

Is the rationale for the Open Letter provided in sufficient detail? (Please consider whether existing challenges in the field are outlined clearly and whether the purpose of the letter is explained)

Partly

Is the Open Letter written in accessible language? (Please consider whether all subject-specific terms, concepts and abbreviations are explained)

Partly

Reviewer Expertise:

Japan, diversity, social inequality, well-being

I confirm that I have read this submission and believe that I have an appropriate level of expertise to confirm that it is of an acceptable scientific standard, however I have significant reservations, as outlined above.

## References

[1] : Digital twins in city planning. *Nat Comput Sci* .2024;4(3) : 10.1038/s43588-024-00606-7 192-199 10.1038/s43588-024-00606-7 38532132

[2] : Rethinking Locality in Japan.2021; 10.4324/9781003032137 10.4324/9781003032137

[3] : From urban modelling to city digital twins – Reflections from the applied urban modelling (AUM) symposia. *Environment and Planning B: Urban Analytics and City Science* .2024; 10.1177/23998083241279601 10.1177/23998083241279601

[4] : Life Course, Happiness and Well-being in Japan. *Abingdon: Routledge* .2017;

[5] : Rural areas between decline and resurgence. Lessons from Japan and Austria. *Beiträge zur Japanologie 46 [Contributions to Japanese Studies 46* .2018;15-26

[6] : Home and Family in Japan Continuity and Transformation. *Routledge* .2011;1-24

[7] : Empowering rural cooperation Effects of agricultural policy intervention on rural social capital. *Routledge* .2020;124-139

# Reviewer report: Reviewer response for version 1

**DOI:** 10.21956/openreseurope.19075.r41721

**Published:** 2024-07-23

**Authors:** Erik Jansen

**Affiliations:** 1 Western Norway University of Applied Sciences Faculty of Health and Social Sciences (Ringgold ID: 366044), Bergen, Hordaland, Norway; 2 School of Social Studies, HAN University of Applied Sciences (Ringgold ID: 6032), Nijmegen, Gelderland, The Netherlands

This article is an accessibly written open letter about a comprehensive research project developing more inclusive communities, and in this component particularly with the help of data-driven approaches and AI. Although I am not that deep into the topic to judge whether factual statements and citations are all correct, the rationale and argumentation appears sound and corresponds with the perspective based on my own position in the field.

 Developing a Digital Twin (DT) of an inclusive community is a very ambitious endeavour, as life in a community can be considered life in its entirety. The authors of this article seem to be well aware of this and provide a comprehensive system of domains that they plan to take into account. As such they combine a highly technical approach with a social science approach to inclusive communities. Moreover, their approach seems robust in that they employed a community wide narrative method to detect which themes matter to the community and combined this with a vast literature analysis. This was done to ensure the validity of the DT to monitor inclusivity at large.

 The three main research actions presented are: 1. to build a Life User Digital Twin; 2. to develop an inclusivity evaluation system; and 3. to develop practices by way of guidelines and demonstrations. What I particularly like about the approach is that these actions are embedded in an (apparently, large-scale) interdisciplinary-based Action Research method. If done properly and authentically, this should enable the research team to work on the transformation with local stakeholders in a collaborative and joint learning curve. However, this will not be an easy process, as from socio-technical studies we know that there will be resistance from the prevailing regime, as the result of routines and materials that work against the social change in communities that one will try to achieve. For this it will be of primary importance to safeguard the critical voice in the action research process. One way to do this could actually be to work in a transdisciplinary way, by including people from the community as co-reserachers and co-developers. Moreover, going through the learning curve together, is part of the transition process itself, so this appears rather at odds with the frame of "rolling-out" such as presented in the closing paragraph. But some more on this below.

 I also have a concrete suggestion for the evaluating and monitoring framework: there is mention that a number of (mental) wellbeing scales will be used. However, what would seem particularly useful in this regard could be an indicator system that is explicitly based on the capability approach (CA) as that approach includes not only individual side of wellbeing, but also the contextual and situational conditions to facilitate it. The CA provides an integral way of looking at wellbeing and also social justice, and therefore could be well-equipped conceptually to capture various kinds of inequities that lead to exclusion in communities. Understanding these mechanisms well is quintessential to fostering wellbeing in communities and societies, and a DT that provides an independent (and therefore trustful) data and information source of good quality could prove a valuable resource for those in positions to act (i.e. persons in political or other positions of power).

 Finally, I do have one worry. Between the lines this project, as with many projects that have a clear technical component, seems to follow a somewhat techno-optimist stance. To follow up on my previous remark on safeguarding the critical voice in the action research process, I would urge the authors to also stay (self-)critical as to what the DT will actually be causing in the community systems (including their system actor stakeholders such as companies and governments). Even if authors themselves stay well aware of the notion that the DT will not be *the* solution to everything in terms of inclusive communities, there is a fair chance that other stakeholders involved will have that conviction and will think and act accordingly. Then, the project could get stuck in a track towards the "technology-will-save-us" paradigm in which tendencies towards gaining more control and eliminating diversity and introducing exclusions of new types lure, while this is actually what authors want to avoid. There can be a tension with using data-technologies, such as DT, between gaining insight and control on the one hand, versus protecting individual freedoms and respecting diversity on the other. And from a normative point of view, particularly the latter, ironically, is what inclusive communities should all be about.

 So to conclude, I think this is an interesting article that lays out and concretizes an approach with a DT to foster inclusive communities. However, to be better balanced for the reader I do think some discussion of the conceptual points raised above is warranted to more clearly position the project in the socio-technical spectrum.

Where applicable, are recommendations and next steps explained clearly for others to follow? (Please consider whether others in the research community would be able to implement guidelines or recommendations and/or constructively engage in the debate)

Partly

Does the article adequately reference differing views and opinions?

Yes

Are all factual statements correct, and are statements and arguments made adequately supported by citations?

Yes

Is the rationale for the Open Letter provided in sufficient detail? (Please consider whether existing challenges in the field are outlined clearly and whether the purpose of the letter is explained)

Yes

Is the Open Letter written in accessible language? (Please consider whether all subject-specific terms, concepts and abbreviations are explained)

Yes

Reviewer Expertise:

Social sciences; sustainability; social practices; action research

I confirm that I have read this submission and believe that I have an appropriate level of expertise to confirm that it is of an acceptable scientific standard, however I have significant reservations, as outlined above.

# Reviewer report: Reviewer response for version 1

**DOI:** 10.21956/openreseurope.19075.r41720

**Published:** 2024-07-23

**Authors:** Barbara Holthus

**Affiliations:** 1 German Institute for Japanese Studies, Tokyo, Japan

This Open Letter introduces a Japanese government initiative, a 5-year program called “Developing inclusive community platforms”. This program aims to improve the well-being of the inhabitants of communities, as well as improve issues of diversity and inclusiveness.

 In a first step, the program conducted narrative interviews as well as a review of more than 1000 academic papers as a foundation to its program. The research methodology is that of “interdisciplinary-based Action Research”. The authors of the Open Letter explain that “a fundamental shift in people’s attitudes and behaviours is crucial” to the success of the elevation of well-being and improved diversity and inclusiveness. How this program is to generate a value shift however seems to remain unclear at this stage.

 The program’s main aim seems to be to generate statistical data in order to measure the degree of inclusivity of a community. How small or large a “community” should be thought of, what the “borders” of a community are, and the fact that communities can significantly vary by region, type, age, gender, and social class of the community members: are these questions the government project has thought of?

 The Letter mentions the use of the “Life User Digital Twin”, which sounds intriguing. This idea is described as a “platform”, unfortunately missing a concrete example of how the platform envisioned here would be put into action to overcome barriers to participation?

 The fact that the findings of this 5-year program are to inform town planning and are to help with community center planning is highly laudable. It will be a challenge to put the data generated by the long-term initiative into concrete action, but it is a good start. This author would have liked to hear more about the size of this project: how many researchers are involved? How many communities are to be studied in-depth? While this seems to be a national government initiative, the implementation of strategies from this presumably will fall into the hands of local governments? And the most important question of all: How much money is allocated for this 5-year program and for putting the findings into action to create happier, more diverse and inclusive communities throughout Japan? This author believes a lot of the success hinges on the willingness to channel a significant sum into this undertaking.

 While the Open Letter does a good job of introducing this governmental initiative, the description remains to a certain way elusive, inconcrete. This might be the case with the project itself and the stage of the project at the time of writing. A critical discussion of this initiative by the authors of the Open Letter however is completely absent – a missed chance. This author would have liked to have seen the authors go beyond the sole description of the government initiative, even if only briefly, such as for example how this project is of relevance to what the authors of the Open Letter are researching.

Where applicable, are recommendations and next steps explained clearly for others to follow? (Please consider whether others in the research community would be able to implement guidelines or recommendations and/or constructively engage in the debate)

Yes

Does the article adequately reference differing views and opinions?

Yes

Are all factual statements correct, and are statements and arguments made adequately supported by citations?

Yes

Is the rationale for the Open Letter provided in sufficient detail? (Please consider whether existing challenges in the field are outlined clearly and whether the purpose of the letter is explained)

Yes

Is the Open Letter written in accessible language? (Please consider whether all subject-specific terms, concepts and abbreviations are explained)

Yes

Reviewer Expertise:

sociology of the family, gender, well-being, rural-urban divide, media, social movements, companion animals and animal rights.

I confirm that I have read this submission and believe that I have an appropriate level of expertise to confirm that it is of an acceptable scientific standard.
